# A Portable and Flexible Self-Powered Multifunctional Sensor for Real-Time Monitoring in Swimming

**DOI:** 10.3390/bios11050147

**Published:** 2021-05-08

**Authors:** Yupeng Mao, Yongsheng Zhu, Tianming Zhao, Changjun Jia, Meiyue Bian, Xinxing Li, Yuanguo Liu, Baodan Liu

**Affiliations:** 1 Physical Education Department, Northeastern University, Shenyang 110819, China; maoyupeng@pe.neu.edu.cn (Y.M.); 2001276@stu.neu.edu.cn (Y.Z.); zhaotm@stumail.neu.edu.cn (T.Z.); 2071367@stu.neu.edu.cn (C.J.); 2001264@stu.neu.edu.cn (M.B.); 2 Foshan Graduate School, Northeastern University, Foshan 528300, China; 3 College of Sciences, Northeastern University, Shenyang 110819, China; 4 Health and Exercise Science Laboratory, Institute of Sport Science, Seoul National University, Seoul 08826, Korea; shinsunglee2021@snu.ac.kr; 5 Sports Training Institute, Shenyang Sport University, Shenyang 110102, China; liuyg@syty.edu.cn

**Keywords:** biosensor, ZnO NWs, piezoelectric effect, self-powered, real-time monitoring

## Abstract

A portable and flexible self-powered biosensor based on ZnO nanowire arrays (ZnO NWs) and flexible PET substrate has been designed and fabricated for real-time monitoring in swimming. Based on the piezoelectric effect of polar ZnO NWs, the fabricated biosensor can work in both air and water without any external power supply. In addition, the biosensor can be easily attached to the surface of the skin to precisely monitor the motion state such as joint moving angle and frequency during swimming. The constant output piezoelectric signal in different relative humidity levels enables actual application in different sports, including swimming. Therefore, the biosensor can be utilized to monitor swimming strokes by attaching it on the surface of the skin. Finally, a wireless transmitting application is demonstrated by implanting the biosensor in vivo to detect angiogenesis. This portable and flexible self-powered biosensor system exhibits broad application prospects in sport monitoring, human–computer interaction and wireless sport big data.

## 1. Introduction

Flexible biosensors have received extensive attention in the field of clinical medicine and exercise rehabilitation, and more advanced nanomaterials have been used to monitor human health-related actions [1,2,3,4,5,6]. Physical exercises can not only prevent diseases, but also assist physical rehabilitation. Among the various sports, swimming can obviously improve cardiopulmonary function and further promote muscle formation. Moreover, swimming is also a self-help skill for humans and the most popular event in the Olympic games. In a swimming competition, athletes need to use their own high-quality skills under the condition of saving the most energy, which enables swimmers to extend the effective stroke time to achieve the best competition performance [7,8,9,10,11]. In swimming training, heart rate detection as a common and effective method is always used to monitor the energy consumption. The force produced by the coordination of arms and legs drives the swimmer forward quickly. The coordination involves the changes in joint angle of the whole body and the time to coordinate the forces (frequency) in the process of swimming [12,13]. Therefore, it is indispensable to monitor the joint angle of the body, motion frequency and the changes in heart rate for promoting the development of swimming skills.

Currently, the methods of swimming skill monitoring include kinematic analysis [9,13], dynamic analysis [14,15] and electromyography (EMG) analysis [16,17]. Among them, kinematic analysis mainly relies on video recording to observe the changes in swimming skills (joint angle and frequency). Dynamic analysis mostly uses joint instruments to test the force of each part on land, while EMG analysis tests the electrical signal of muscles. However, these regular monitoring methods also encounter some inevitable bottlenecks in the process of continuous development. For example, multiple cameras need to be arranged in water and underwater to collect the motion angle and frequency information in kinetic analysis [18,19] and skill in swimming needs multi-joint coordination. In this process, the position and direction of the joints are constantly changing. In order to observe their motion state, it may be necessary to arrange multi-point cameras to capture them. Therefore, monitoring is difficult and the cost is high. The lengthy recording with multiple cameras also leads to high power consumption and increases the difficulty of testing. The dynamic and EMG tests on land are also composed of multiple devices, which are very huge and cannot be used in water, increasing the complexity in dynamic swimming and making it impossible to achieve portable applications. In recent years, there have been many studies on portable human motion monitoring sensors, most of which were carried out in air [20,21]. The application of water sensors involves studies monitoring water quality [22,23]. Therefore, it is extremely urgent to develop portable, self-powered and efficient hydrophilic biosensors that are not limited by batteries and volume to precisely and quickly monitor exercise skills and heart rate changes in the future [24,25,26,27,28,29,30].

In this work, we report the design and integration of a self-powered, flexible biosensor based on ZnO nanowire arrays (NWs) with inter-digital electrodes. Based on the piezoelectric effect of polar ZnO, the biosensor can harvest the mechanical energy coming from body activity to drive the biosensor to monitor the motion process in air and water. In this way, the motion information and swimming skills such as the change in joint angle and motion frequency can be monitored in real time. Moreover, this study also simulates a sensor that can be attached to the big artery to detect the change in heart rate and the signals can be further transmitted based on conventional wireless technology. Therefore, the developed work and related technology of the biosensor show significant application prospects in sport monitoring, human–computer interaction and infinite sport big data.

## 2. Experimental

### 2.1. Fabrication of Self-Powered Biosensor

First, the ZnO NWs were synthesized on a PET substrate by a simple hydrothermal method, as reported in our previous work [29,30,31,32,33,34]. A 2.2 mL ammonia solution was gradually added to a 38 mL solution containing 0.8 g zinc nitrate and heated at 93 °C for 24 h. Then, the obtained vertical ZnO NWs were transferred to flexible PET substrate. Thirdly, a photolithography process and electron beam evaporation process were used to deposit the Ti electrode on both sides of ZnO NWs. The thickness of the photoresist and Ti electrodes was ~2 μm and ~200 μm, respectively. Typically, the length of ZnO NWs was more than ~10 μm to fully cover the electrode pairs (5 μm).

### 2.2. Characterization and Measurement

The morphology of the ZnO NWs was studied by a scanning electron microscope (SEM, JEOL JSM-6700F). The crystalline phase of ZnO NWs was characterized by X-ray diffraction (XRD, D/max 2550 V, CuKa radiation). It should be pointed out that the biosensor was placed in an open container for standard measurements and the performance on human skin was finally tested in a swimming pool. A low-noise preamplifier (Model SR560, Stanford Research Systems) was used to measure the piezoelectric output.

## 3. Results and Discussion

**Figure 1:**
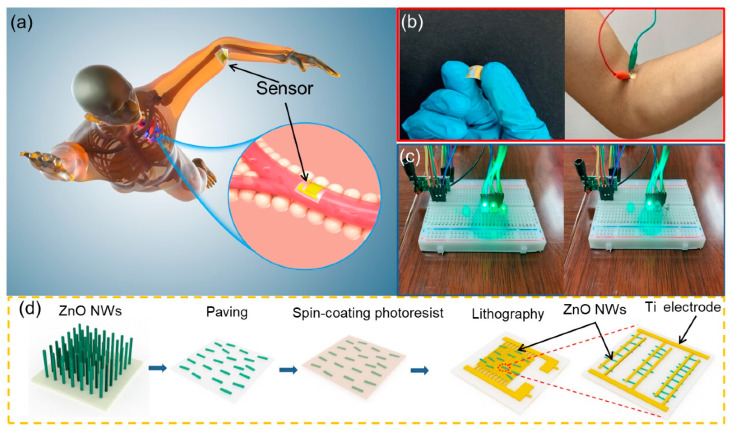
A potential scenario and the process of synthesis of self-powered biosensor in swimming monitoring. (**a**) Simulating the monitoring of athlete’s elbow joint angle and heart rate. (**b**) Optical image of biosensor. (**c**) Simple wireless transmitter and information receiver. (**d**) Process of synthesis of self-powered biosensor.

Figure 1 shows the experiment design, potential scenario and the schematic fabrication process of a self-powered biosensor in swimming monitoring. The fabrication process of the biosensor and the details of the electrode and ZnO NWs are shown in Figure 1d. Firstly, the length of vertically aligned ZnO is controlled in the range of 5–15 μm by a hydrothermal method to make sure that it can fully cover the neighboring electrodes. As schematically illustrated in Figure 1a, the flexible self-powered biosensor can be easily attached to the tester’s joint in in vivo monitoring. Due to the piezoelectric effect of polar ZnO NWs, the biosensor can convert motion energy (produced by the human body in motion) into electric energy for self-driving, and work in high humidity or water, providing more potential application scenarios such as heart rate monitoring, organ monitoring and blood vessel wall monitoring. In the biosensor, the bioinformation can also be transformed by a simple Bluetooth system to monitor and collect the data, including moving frequency, the change in angle and physiology information, and thus help to formulate sport plans or adjust sport skills. Figure 1b shows the optical images of the self-powered biosensor and the attached position of the three swimming styles, and the optical micrographs of commercial LEDs connected with the biosensor that is attached to a tester’s elbow joint. A simple Bluetooth wireless transmission system using a commercial signal amplification device is used as the launcher and receiver of Bluetooth in Figure 1c. It can be seen that with the increase in the output of piezoelectric voltage of the biosensor, the number of LED lamps that are turned off at the receiving end increases.

**Figure 2:**
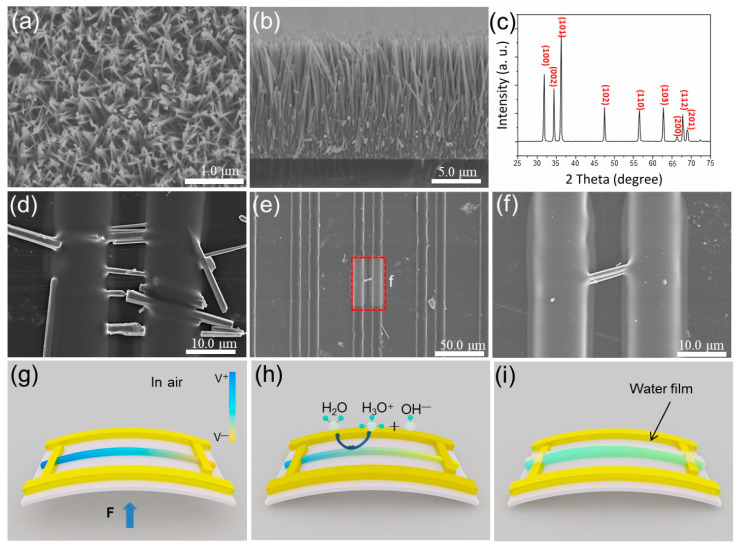
(**a**) Top view SEM image of ZnO. (**b**) Side view SEM image of ZnO. (**c**) XRD image of ZnO. (**d**) SEM image of ZnO NWs crossing a pair of electrodes in the same direction. (**e**) SEM image of a single ZnO NW crossing a pair of electrodes. (**f**) High-magnification morphology of a single ZnO NW crossing a pair of electrodes (Figure 2e). (**g**) Working mechanism of ZnO NW. (**h**) Working mechanism of ZnO NW under low-humidity conditions. (**i**) Working mechanism of ZnO NW under high-humidity conditions.

Figure 2 shows the SEM images and XRD pattern of ZnO NWs synthesized from a hydrothermal process and the ZnO-based biosensor, as well as its schematic diagram of the piezoelectric process. It can be seen that ZnO NWs grow in the same direction and show good alignment (Figure 2a). The length of vertical ZnO crystals is about 5–10 μm, which is enough to cover the distance between electrodes (Figure 2b). Figure 2c shows the XRD pattern of ZnO NWs, inserted in the right top corner, and the sharp diffraction peaks indicate good crystalline quality. Figure 2d–i are the SEM images of ZnO nanowires on both ends of a titanium electrode. As shown in Figure 2d, ZnO nanowires are arranged in the same direction on the PET substrate. It provides an opportunity to evaporate Ti electrodes on both ends of ZnO (using photolithography). Figure 2e shows one ZnO NW crossing a pair of electrodes, and Figure 2f is an enlarged view of Figure 2e. We can confirm that the ZnO NWs are well linked with the electrodes to ensure the good transfer of electrons generated by the piezoelectric effect. Figure 2g shows the working mechanism of the ZnO NW. When the nanogenerator is bent under deformation, the two ends of the ZnO NW will provide piezoelectric potential. Figure 2h shows the working mechanism of the ZnO NW under low-humidity conditions. When the ZnO NW is exposed to a humid environment, a large number of oxygen vacancies on the surface of the ZnO NW will adsorb the water molecules in the air [31,32,33,34,35,36,37], and the chemical adsorption will produce ionization [38,39,40,41]. The H2O molecules dissociate into hydronium ions and hydroxide ions, which enhances the shielding effect, reducing the piezoelectric output. Additionally, as shown in Figure 2i, with the humidity continuously increasing, all the oxygen vacancies on the surface of the ZnO NWs are occupied by water molecules and form a water film. The shielding effect is saturated, so the piezoelectric voltage will remain constant in spite of the change in humidity.

**Figure 3:**
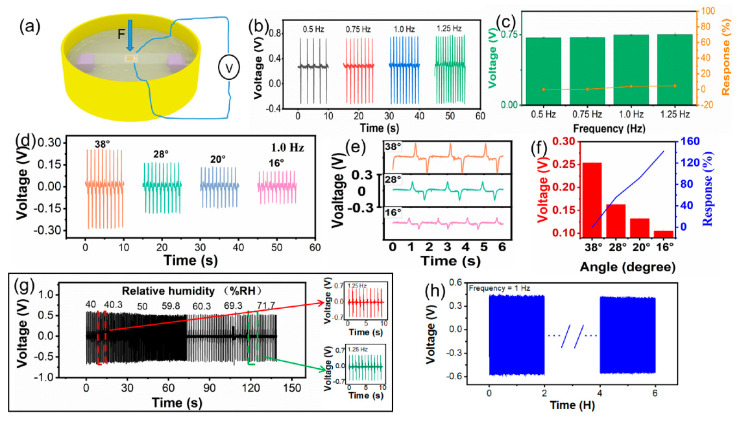
Experimental control of the self-powered biosensor. (**a**) Simulated experimental control of testing environment. (**b**) Output piezoelectric voltage at different motion frequencies. (**c**) Output piezoelectric voltage response at different frequencies. (**d**) Output piezoelectric voltage at the same frequency and different angles. (**e**) Details of output piezoelectric voltage of angle variation. (**f**) Output piezoelectric voltage response of angle variation. (**g**) Output piezoelectric voltage at different humidity levels. (**h**) Biosensor durability.

Figure 3 shows the self-powered biosensor working under different simulated environments and experimental controls. It can be seen that the self-powered biosensor works in different modes of air and liquid, and experimental controls show a different response to the change in humidity of the test environment or the test in water (Figure 3a). The output piezoelectric voltage of the biosensor generated at different motion frequencies is shown in Figure 3b. We can see that the output piezoelectric voltage is 0.718, 0.72, 0.731 and 0.735 V, respectively, when the biosensor is bent at 0.5, 0.75, 1.0 and 1.25 Hz at the same bending angle. The world record of the 50 m men’s freestyle in a short pool is 20 ’’ 91. The number of arm strokes of the world’s top male athletes is about 17–20 (from a video of the Olympic Games), so we tested the performance under 5 Hz (in Figure S1). It showed that the sensor can still output a stable piezoelectric signal at a high frequency, which can meet the needs of swimming monitoring. The relationship between output piezoelectric voltage and deformation at different frequencies is shown in Figure 3c. The response of the biosensor can be calculated with the following equation:

(1) $$R\%=\left|\frac{V_{0}-V_{i}}{V_{i}}\right|\times 100\%,$$

where *V*_0_ is the output piezoelectric voltage under 0.5 Hz, and *V_i_* is the output piezoelectric voltage under other frequencies. When the biosensor is bent at 0.5, 0.75, 1.0 and 1.25 Hz at the same bending angle, the corresponding response of the piezoelectric output is 0, 2, 2.2 and 2.2%. However, the output piezoelectric voltage of the biosensor is hardly changed with the variation in motion frequency, as shown in Figure 3b. From the above result, it can be seen that the output frequency of the piezoelectric voltage increases with the bending frequency, so the performance of the ZnO NW biosensor can be used to monitor the frequency change in the human body (joint). The output piezoelectric voltage of the biosensor at the same frequency and different bending angles is shown in Figure 3d. When the bending angle is set at 38, 28, 20 and 16°, the corresponding output piezoelectric voltage of biosensor is 0.254, 0.163, 0.132 and 0.105 V, respectively. The details of the output piezoelectric voltage of the biosensor as a dependency of the bending angle are plotted in Figure 3e. It can be seen that the larger the bending angle (the greater the deformation of the biosensor) is, the greater the output piezoelectric voltage is. Meanwhile, the response of the biosensor is also proportional to the variation in the bending angle. The *V*_0_ is the output piezoelectric voltage under 38° and *V_i_* is the output piezoelectric voltage under other bending angles. When the bending angle is set at 38, 28, 20 and 16°, the corresponding response is 0, 55.8, 92.4 and 141.9% (Figure 3f). The output piezoelectric voltage of the biosensor under deformation also shows a similar tendency as when it is bent under different angles, so it can also be used to monitor the motion angle of the human body (joint). To explore more potential applications, we tested the output piezoelectric voltage of the ZnO NW biosensor under different humidity conditions, as shown in Figure 3g. The output piezoelectric voltage of the biosensor is steady in regular conditions and a humidity level of more than 45%. This is because a large number of oxygen vacancies on the surface of the ZnO NWs adsorb water molecules [31,32,33,34] when the biosensor is exposed to a humid environment. Additionally, chemical adsorption also produces ionization [36,37,38,39,40,41] which in turn enhances the shielding effect, so the piezoelectric voltage of the ZnO NWs decreases under the same deformation conditions. When the humidity increases to 60%, the piezoelectric voltage remains stable because the oxygen vacancies on the surface of the ZnO NWs are occupied by water molecules, forming a thin water film. Then, the shielding effect does not continue to increase with the increasing humidity [25,42,43,44,45,46,47]. Furthermore, the piezoelectric voltage is not affected by the change in humidity, so the biosensor can be tested and used to monitor a swimmer in water. In our previous work, we discussed the sensing response of ZnO modified by lactate, and the unmodified ZnO had no sensing performance on sweat [5,44,45]. Sweat appears with swimming and it may be rapidly diluted by water, which may have few effects on our sensor. Therefore, the effects of sweat are not discussed in this paper. From Figure 3h, we can see that the output piezoelectric voltage of the biosensor remains stable after 6 h of continuous operation, indicating its good stability. The above data demonstrate that the designed biosensor has potential applications in sensing motion frequency, angle and humidity, and has a superior waterproof function, contributing toward reliable working life.

**Figure 4:**
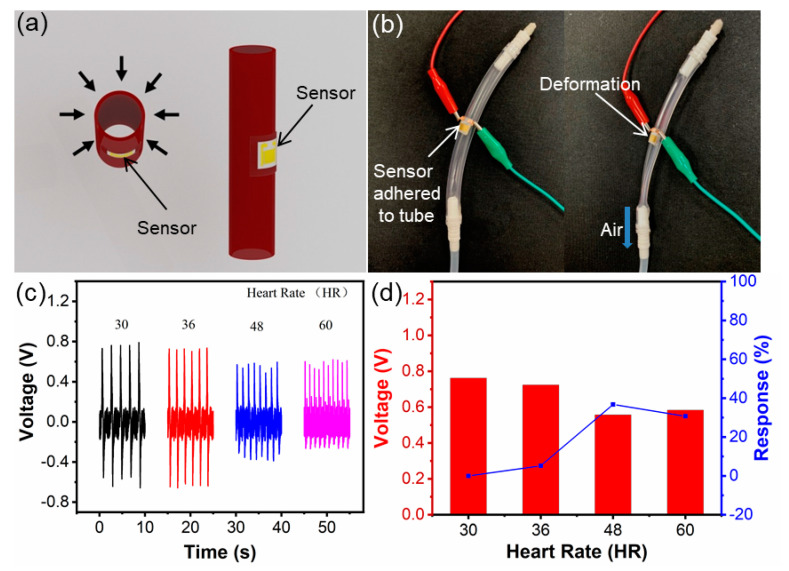
Photo of simulated arterial biosensor and characterization of output piezoelectric voltage. (**a**) Simulation of arterial work and biosensor attachment position. (**b**) Arterial model. (**c**) Output piezoelectric voltage of the arterial model monitored by biosensor. (**d**) Output piezoelectric voltage response.

Figure 4 shows the schematic diagram of the ZnO NW biosensor for simulating an arterial sensing test in vitro and the output piezoelectric voltage, and a potential application scenario for monitoring the heart/internal aortic vessel is proposed. The application scenario of the biosensor is designed so that it can be attached to the blood vessel to monitor the pulse vibration (Figure 4a). As can be seen, the biosensor is attached to the simulated artery, and when the air is pumped in or out, the flexible biosensor deforms according to the simulated artery model shown in Figure 4b. In this way, the output piezoelectric voltage is produced by the biosensor when monitoring different pulse frequencies and the amplitudes of simulated arteries are shown in Figure 4c. When the pulse frequency is set at 30, 36, 48 and 60 times per minute, the output piezoelectric voltage is 0.761, 0.724, 0.557 and 0.583 V, correspondingly. We can clearly observe that the output frequency of the piezoelectric voltage changes with the increase in pulse frequency, and different deformations of the simulated arterial surface are caused by different pulses (pumping out air often leads to large surface deformation of the simulated artery and slow pulse frequency). This can be further verified by the characterization of the output piezoelectric voltage of biosensor. The output response of the piezoelectric voltage is 0, 5, 36.8 and 30.7% when the pulse frequency of the simulated artery is fixed at 30, 36, 48 and 60 times per minute (Figure 4d). Through the application of wireless transmission, the concept of degradable flexible biosensors for pulse or deformation monitoring of the heart and organ surfaces can be applied to more scenarios, such as vascular surgery, early detection of vascular failure [1,2,3], etc.

**Figure 5:**
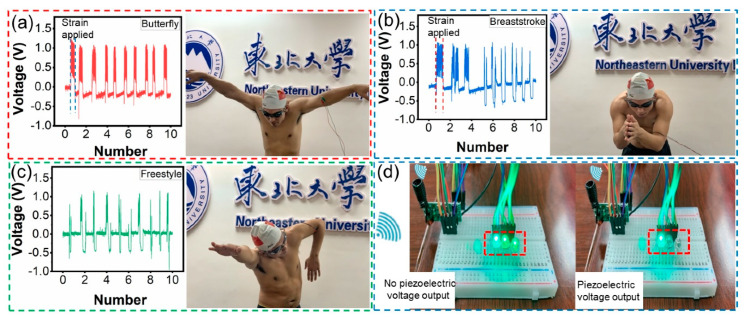
Experimental design of integrated wireless transmitter and receiver for biosensor. (**a**) Output piezoelectric voltage of butterfly stroke. (**b**) Output piezoelectric voltage of breaststroke. (**c**) Output piezoelectric voltage of freestyle stroke. (**d**) Wireless transmission analysis station.

Figure 5 shows the practical application scenario of a biosensor integrated with a wireless transmitter receiver. The sensor can be easily attached to the inside of the elbow joint between the upper arm and forearm. The rhythmic motions of the arm skills in butterfly stroke are shown in Figure 5a. The output piezoelectric voltage of the biosensor is 1.272, 1.086, 1.072, 1.079, 1.04, 1.067, 1.062, 1.099, 1.086 and 1.084 V. The arm skills of butterfly stroke are divided into four parts: entering, holding, sculling and pushing [9,10,11,12,13]. The output piezoelectric voltage of the biosensor is relatively stable, which indicates that the tester’s arm skills are relatively stable in each butterfly stroke part. It is worth noting that the peak’s width of the piezoelectric voltage in Figure 5a is slightly wider (within the blue dotted line in Figure 5a). It is assumed that this can be ascribed to the strain applied to the sensor. This part is the technical connection process of the arm from holding to sculling and then to pushing. To verify our claim, we monitored the arm skills of breaststroke, as shown in Figure 5b. The arm skills of breaststroke include five parts: sliding, holding, sculling, hand closing and arm extension. The tester intends to extend the time of hand closing and arm extension in the first three breaststrokes. At this time, we can observe that the angle between the upper arm and forearm is the smallest, while the deformation of the biosensor is the largest, and the output piezoelectric voltage is the highest. After that, the peak width of the output piezoelectric voltage widens (in the red dotted line in Figure 5b) after deliberately extending the hand closing and arm extension. It is believed that the strain applied to the biosensor induces the deformation due to the results of the micro change in the same bending angle of the device. The output piezoelectric voltage of ten breaststroke arm skills is 1.079, 1.042, 0.942, 1.025, 0.95, 1.079, 0.967, 1.064, 0.942 and 0.847 V under stable conditions. For freestyle arm skills, they are generally divided into two main parts: in water and out of water. The action of arms in the water includes entering water, catching water, pulling water and finally pushing water, then immediately becomes out of water action and movement in the air. The output piezoelectric voltage of the freestyle arm skills of the tester is also relatively stable in Figure 5c. By observing the output piezoelectric voltage signal, we can see that the frequency and angle are quite stable, and the performance of motion skills is extremely good. The duration of different swimmers’ arm skills is different in each part, and most swimmers are looking for a suitable swimming skill to adjust the frequency and angle of arm skills. Unfortunately, the collection equipment cannot adapt to the wet environment of the swimming pool. In order to prevent the occurrence of unpredictable risks, the above complete swimming skills are not monitored underwater. However, a substitute experiment is conducted to exhibit the potential application in swimming training. In Movie S1, when the sensor is put into the swimming pool water, we can see that the sensor still has piezoelectric output. It shows that the sensor has the potential to be used in underwater swimming skills monitoring. In this way, flexible biosensors can be portable, non-destructive and used in real time to monitor the frequency, angle and other information of swimming skills, so as to help make training plans and improve sport performance. As a result, the data can be analyzed by a simple wireless transmitting and receiving station, as shown in Figure 5d. The output piezoelectric voltage generated by the body motion of the tester is charged to a 4.7 μf capacitor device, and the voltage of 0.72 V (Figure S2) is charged within 80 s. After charging, the wireless information transmission is driven and sent to the receiving station. Movie S2 shows the experimental process. When there is no output piezoelectric voltage, the LED lamps in the receiving station cannot be turned off. When the piezoelectric output increases or the frequency changes, the receiving station turns off different numbers of LED lamps or they flash at the same frequency. Such wireless information transmission functions provide more potential application scenarios for sport big data transmission.

## 4. Conclusions

In summary, a portable self-powered biosensor based on the electromechanical coupling effect of ZnO NWs has been designed and demonstrated to monitor various swimming styles of swimmers. This work also demonstrates the potential application of ZnO NW biosensors in a simulated scenario of monitoring the aortic pulse and realizing real-time wireless transmission. The biosensor perceives the human body’s motion frequency, joint angle and underwater performance through wireless transmission, and uses the information from the receiving station to actively monitor the swimmer’s performance. This type of flexible biosensor possesses broad application prospects in human–computer interaction monitoring, infinite sport big data transmission and self-powered portable monitoring systems.

## Data Availability

The data presented in this study are available in [insert article or supplementary material here].

## Supplementary Materials

The following are available online at https://www.mdpi.com/article/10.3390/bios11050147/s1. Figure S1: Capacitor charge. Figure S2: Output piezoelectric voltage under 5 Hz. Movie S1: Numbers of LEDs driven by the self-powered sensor under water. Movie S2: LEDs controlled by self-powered sensor.
