# Supplementary material for: A Portable and Flexible Self-Powered Multifunctional Sensor for Real-Time Monitoring in Swimming
Source: Biosensors (Basel). 2021 May 8;11(5):147. doi: 10.3390/bios11050147 (PMC8151083; doi:10.3390/bios11050147)
Supplement: Supplementary file 1 [file biosensors-11-00147-s001.zip › Supplementary Materials -1.pdf]

A Portable and Flexible Self-Powered Multifunctional Sensor for Real-Time Monitoring in Swimming

Yupeng Mao [1], Yongsheng Zhu [1], Tianming Zhao [1,2,3], Changjun Jia [1], Meiyue Bian [1], Xinxing Li [4], Yuanguo Liu [5] and Baodan Liu [1,2,*]

[1] Physical Education Department, Northeastern University, Shenyang 110819, China; maoyupeng@pe.neu.edu.cn (Y.M.); 2001276@stu.neu.edu.cn (Y.Z.); zhaotm@stumail.neu.edu.cn (T.Z.); 2071367@stu.neu.edu.cn (C.J.); 2001264@stu.neu.edu.cn (M.B.)
[2] Foshan Graduate School, Northeastern University, Foshan 528300, China
[3] College of Sciences, Northeastern University, Shenyang 110819, China;
[4] Health and Exercise Science Laboratory, Institute of Sport Science, Seoul National University, Seoul 08826, Korea; shinsunglee2021@snu.ac.kr
[5] Shenyang Sport University, Shenyang 110102, China
* Correspondence: liubaodan@mail.neu.edu.cn (B.L.)

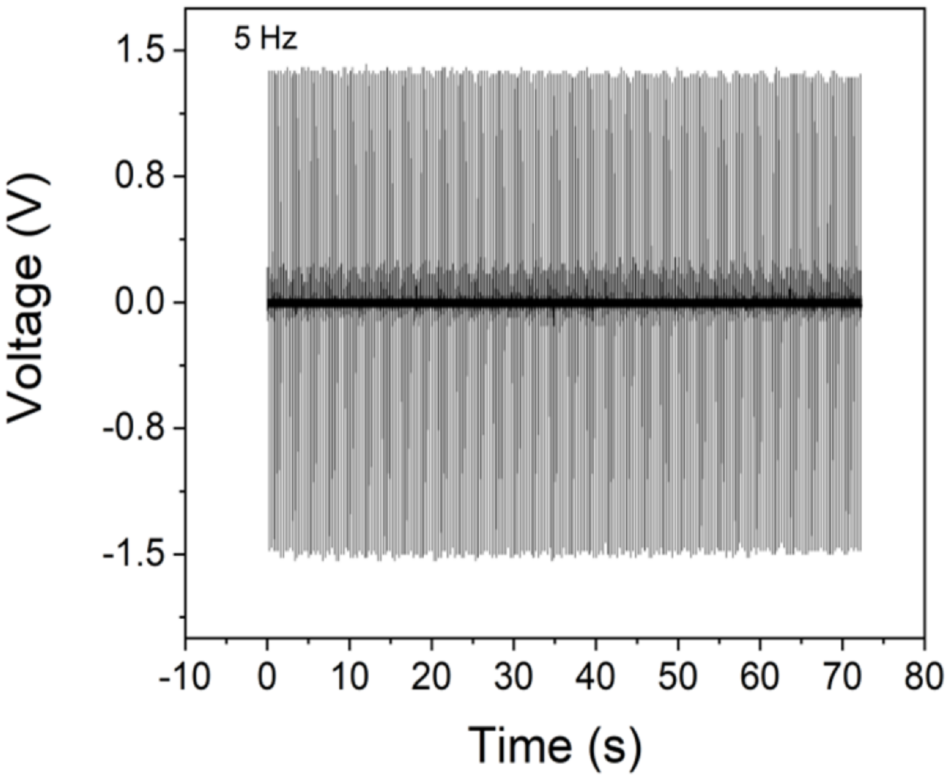

Figure S1. Output piezoelectric voltage under 5 Hz.

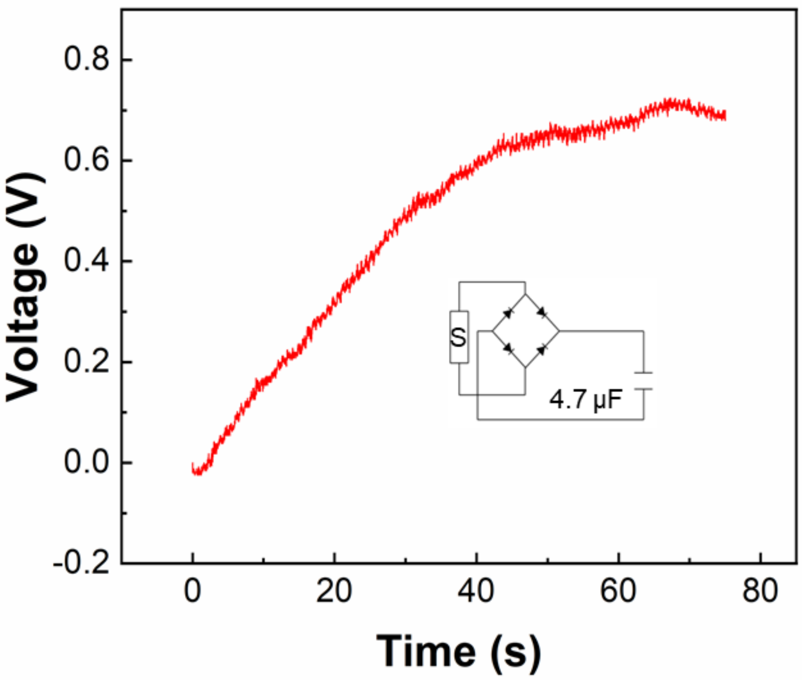

Figure S2 Capacitor charge.

Supplementary Movie S1. Numbers of LEDs driven by the self-powered sensor under water. The times and the numbers of LEDs on the visual panel, which are lit up, can reflect the sport states.

Supplementary Movie S2. LEDs controlled by self-powered sensor. LEDs are contacted with a wireless receiver and the self-powered sensor is contacted with a wireless transmitter. LEDs can be controlled by self-powered sensor/
